# Early Diagnosis of Oral Squamous Cell Carcinoma Based on Histopathological Images Using Deep and Hybrid Learning Approaches

**DOI:** 10.3390/diagnostics12081899

**Published:** 2022-08-05

**Authors:** Suliman Mohamed Fati, Ebrahim Mohammed Senan, Yasir Javed

**Affiliations:** 1 College of Computer and Information Sciences, Prince Sultan University, Riyadh 11586, Saudi Arabia; 2 Department of Computer Science & Information Technology, Dr. Babasaheb Ambedkar Marathwada University, Aurangabad 431004, India

**Keywords:** CNN, ANN, SVM, hybrid method, OSCC, FCH, DWT, LBP, GLCM

## Abstract

Oral squamous cell carcinoma (OSCC) is one of the most common head and neck cancer types, which is ranked the seventh most common cancer. As OSCC is a histological tumor, histopathological images are the gold diagnosis standard. However, such diagnosis takes a long time and high-efficiency human experience due to tumor heterogeneity. Thus, artificial intelligence techniques help doctors and experts to make an accurate diagnosis. This study aimed to achieve satisfactory results for the early diagnosis of OSCC by applying hybrid techniques based on fused features. The first proposed method is based on a hybrid method of CNN models (AlexNet and ResNet-18) and the support vector machine (SVM) algorithm. This method achieved superior results in diagnosing the OSCC data set. The second proposed method is based on the hybrid features extracted by CNN models (AlexNet and ResNet-18) combined with the color, texture, and shape features extracted using the fuzzy color histogram (FCH), discrete wavelet transform (DWT), local binary pattern (LBP), and gray-level co-occurrence matrix (GLCM) algorithms. Because of the high dimensionality of the data set features, the principal component analysis (PCA) algorithm was applied to reduce the dimensionality and send it to the artificial neural network (ANN) algorithm to diagnose it with promising accuracy. All the proposed systems achieved superior results in histological image diagnosis of OSCC, the ANN network based on the hybrid features using AlexNet, DWT, LBP, FCH, and GLCM achieved an accuracy of 99.1%, specificity of 99.61%, sensitivity of 99.5%, precision of 99.71%, and AUC of 99.52%.

## 1. Introduction

Oral cancer is the growth of abnormal cells in the oral cavity, which cannot be controlled in its late stages. Among the oral cancers, oral squamous cell carcinoma (OSCC) is the most common oral malignancy that originates in the oral cavity [1,2], which occurs when multiple genetic mutations accumulate within the cells [2], resulting in damage to the epithelium. Although it begins to appear in the oral epithelium. As a result, such cells and the associated nucleus change in size and shape. Particularly, there are three different grades of OSCC, namely well-differentiated OSCC, moderately well-differentiated OSCC, and poorly differentiated OSCC. According to the Public Health Organization reports, OSCC is the seventh most common type of cancer worldwide [3], with an annual global incidence of 657,000 people and approximately 330,000 deaths. OSCC is associated with several risk factors such as tobacco and alcohol use, poor oral hygiene, infection with human papillomavirus (HPV), ethnicity, geographic location, and family history. The danger of OSCC is in the fact that there are no specific clinical vital signs that help experts accurately predict OSCC. However, it can be predicted by many indicators such as the location of the lesion inside the mouth, its color, size, appearance, and tobacco and alcohol use [4]. Alternatively, expert pathologists diagnose OSCC by observing biopsy strips taken from cells or tissues of the tumor area. Microscopic examination of biopsy slides is considered the gold standard for the effective diagnosis of carcinoma. In this case, experts take a small portion of the tumor, prepare slides stained with hematoxylin and eosin (H&E), and analyze them under a microscope. However, this method is laborious, requires high expertise, is time-consuming, and is prone to errors. Thus, the need for early diagnosis of OSCC is essential for effective treatment, increasing the chances of survival and reducing the mortality rate. In OSCC stage IV, the five-year survival rate is 20:30%, while the early-stage (first) survival rate is 80% [5]. Computer-aided systems are indispensable to automatically distinguishing benign cells from malignant cells of OSCC based on the features of each tumor with better accuracy. In addition, introducing artificial intelligence techniques, particularly machine learning and deep learning, to improve diagnosis is promising. Although time-consuming, deep learning techniques have demonstrated their ability to diagnose and analyze biomedical images accurately. Conventional neural network (CNN) models are one of the best deep learning methods for this purpose. By comparing the features of each new image (test) with the features in the stored (training data), the CNN models are first trained to know the features of each disease. After the training, the CNN model will be able to predict the unknown cases. However, CNN models’ accuracy is affected by several factors such as noise in data set images, a lack of data sets, unbalanced data sets, the number of layers used, activation function, and others. Hence, this study aimed to investigate such challenges facing CNN models and tried to address them to reach superior results in histology diagnosis, which is critical for early diagnosis of OSCC. To achieve this study’s aim, the data set images were enhanced to remove noise and address time-consuming problems and the requirement of costly computers using hybrid techniques between deep learning and machine learning. Moreover, diagnoses of OSCC were based on fusion features between deep learning models and features of color, texture, and shape extracted by the DWT, LBP, FCH, and GLCM algorithms.

The main contributions of this study are as follows:

- Two overlapping filters were applied to improve histological images of oral cancer.
- Effective diagnosis of histological images of oral cancer cells using a hybrid technique between CNN models and the SVM algorithm.
- The PCA algorithm was applied to reduce the dimensionality of the elevated OSCC data set features.
- Diagnosing the histological images of oral cancer cells using the ANN algorithm based on the hybrid features extracted by CNN models and combining them with the color, texture, and shape features extracted by the DWT, LBP, FCH, and GLCM algorithms.
- Designing high-efficiency systems to help specialist doctors in making accurate diagnostic decisions.

The remainder of this paper is arranged as follows: A set of previous studies is presented in Section 2. An investigation of the materials and techniques used for the analysis and interpretation of the histological images of OSCC diagnostic methodologies is presented in Section 3. The results of the evaluation of the proposed methods are described in Section 4. The discussion and comparison of the approach used in this study are provided in Section 5. The conclusion of this study is presented in Section 6.

## 2. Related Work

In this section, a critical review of the most relevant studies in the literature is presented to shed the light on OSCC diagnosis trends and challenges. As observed throughout our review, each researcher aims to reach a promising diagnostic accuracy using different methods.

Ibrar et al. [6] presented conversion learning based on adapting deep learning to diagnose histopathological images to diagnose OSCC. They extracted and categorized deep features using three CNN models; the models achieved an accuracy of 89.16% with VGG16. Tabassum et al. [7] discussed the methodology of examining structures for oral cancer diagnosis through microscopic biopsy images. The lesion area was manually segmented, and then the structural and morphological features were extracted for further analysis. Features were fed to five machine learning classifiers. Seda et al. [8] developed a technique for classifying histopathological images as suspicious or normal based on learned transmission models and creating heat maps to focus on an area of interest. Two data sets from the United Kingdom and Brazil were diagnosed by cross-validation and leave-one-patient-out verification. The method achieved an accuracy of 73.6% and 90.9% for both data sets. Tabassum et al. [9] presented a method for diagnosing histopathological images by extracting shape, color, and texture features. The features were fed into the decision tree, logistic regression, and SVM classifiers. SVM achieved the best performance for classifying color and texture features. Veronika et al. [10] presented a MobileNet model for diagnosing squamous cell carcinoma through pooled samples of 20 patients. The model achieved a sensitivity and specificity of 47% and 96%, respectively. Bishal et al. [11] presented a CNN model with a loss function to reduce the error rate in diagnosing oral tumors and increase the accuracy of diagnosis in less treatment time. The system was trained and tested on an oral tumor data set. The system achieved an overall accuracy of 96.5% while reducing processing time. Francesco et al. [12] presented four methodologies based on deep learning for oral cancer lesion segmentation. The Cancer Genome Atlas data set was segmented for training and testing to evaluate image segmentation models for training and testing. The methods achieved good results in lesion pixel segmentation. Martin et al. [13] proposed a spectroscopic method based on reflections and auto imaging to diagnose SCC at the margins of cancer for 102 patients and compare it with fluorescent dyes. Deep learning models were evaluated on a new data set of 293 patients for SCC detection. The system was evaluated using the AUC scale, which achieved 82% with a margin of 3 mm around cancer. The study proved that the imaging based on reflection and self-performance outperforms the proflavine dye in the RGB color system. Jelena et al. [14] proposed a two-stage method for automatic classification and segmentation of stromal and epithelial tissues for histopathological images of oral cancer. The integrated system Xception and SWT achieved the best rating of 96.3%. Alberto et al. [15] presented a fully CNN for semantic segmentation of SCC in the oral cavity and pharynx. Two data sets were diagnostic for analyzing 34 and 45 video clips of the oral and pharyngeal lesions. 110 and 116 frames were extracted from the video for both oropharyngeal lesions. Three models of FCNNs were applied to the segmentation of tumors. ResNet achieved the best performance as the dice coefficient reached 65.59% and 76.03% for oral and pharyngeal data sets, respectively. Santisudha et al. [16] proposed a capsule network model based on deep learning to classify malignant tumors in the oral cavity. The capsule mesh was applied in agreement with consensual and dynamic routing to make it more robust for afferent rotation and affine transformation to analyze histopathological images of OSCC with high accuracy. Andrés et al. [17] presented a method for predicting nodular malignancy in oral cancer by machine learning. Algorithms were evaluated on a data set of nodular malignancies from 782 patients. The results of the proposed algorithms were compared with a depth-of-invasion-based model by Delong of the AUC curve. The decision set algorithm achieved the best AUC performance of 84%. Mingxin et al. [18] presented CNN and Raman spectroscopy models to distinguish tongue cancer from non-neoplastic tissue. From the Raman spectra, non-linear features were extracted by six blocks, each block having a convolutional layer and a max-pooling layer. The features were fed to fully connected layers to classify them. Rachit et al. [19] presented CNN models to classify 672 histological images of dysplasia of the epithelial layer of the oral cavity from 52 patients. Images were enhanced, and data augmentation was performed to overcome the problem of overfitting. Deep feature maps were extracted and categorized; the model reached an accuracy of 89.3%.

According to the above discussion, all the presented work focused on pre-trained deep learning models or machine learning algorithms. Thus, this current study aimed to develop hybrid systems between deep learning and machine learning models and hybrid methods for extracting features through deep learning models and integrating them with features of color, texture, and shape. This hybridization will help build highly efficient systems for diagnosing the OSCC, leading to promising accuracy.

## 3. Materials and Methods

**Figure 1:**
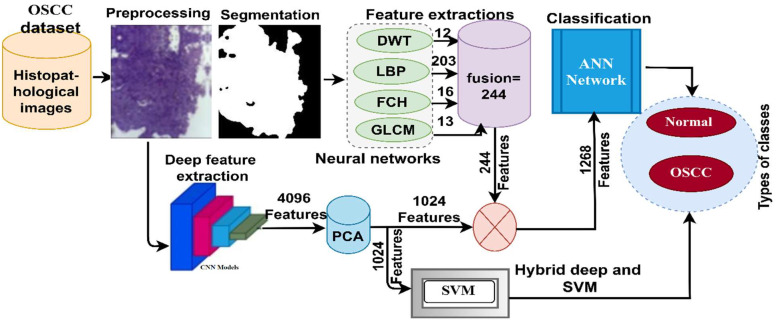
Structure of the histopathological image diagnostics methodology for early diagnosis of OSCC.

This section reviews the materials and methodologies used in this study for classifying histopathological images for early diagnosis of OSCC, as depicted in Figure 1. As the OSCC data set images contained artifacts, the first step was to optimize all histological images. To achieve this study’s aim, there were two approaches deployed with two systems for each. The first approach was based on a hybridization of both CNN models and SVM algorithms, while the second approach was to diagnose data set OSCC by the ANN based on hybrid features extracted by CNN models with color, shape, and texture features extracted by the DWT, LBP, FCH, and GLCM algorithms.

### 3.1. Data Sets

**Figure 2:**
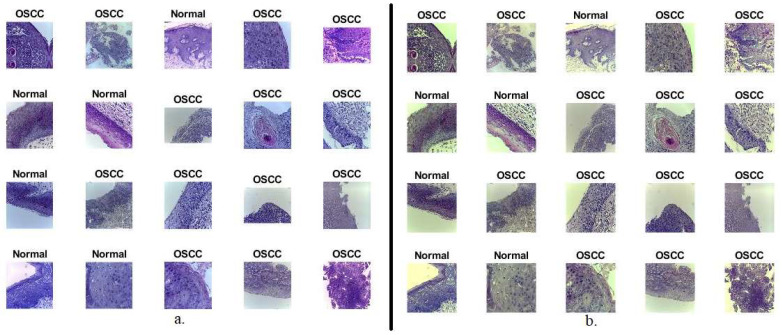
Samples of histopathological images of OSCC. (**a**) Original images before the enhancement process; (**b**) optimized images after the enhancement process.

In this study, the proposed systems were evaluated on a histopathological image of the data set OSCC, which is a public data set. The data set includes 5192 histopathological images taken from biopsy slides with a 100× magnification. All data set images were taken by biopsy with a local anesthetic. The biopsies were diagnosed by a pathologist, and the images were obtained by a magnification technique under the microscope with up to 100× magnification. The data set was divided into 2494 normal histopathological images representing 48% of the total images and 2698 malignant histopathological images of OSCC representing 52% of the total images. Normal images in the data set were determined to be non-cancerous tissue after analysis by a pathologist. The histopathological images, the focus of this study, contained the squamous epithelial layer, connective tissue, and adipose tissue. Figure 2a describes a set of data set samples for the two classes [20].

### 3.2. Preprocessing of Histopathological Images

Preprocessing is one of the most critical steps in biomedical image processing, which helps to coordinate images appropriately to obtain high accuracy. CNN models require expensive computations and consistent formatting of input images. The biopsy slides contain dark areas, and some of them are stained with blood and some medical solutions; therefore, there is a difference in the color of the images of the slides. Thus, the average RGB color for each image was calculated; then, the color consistency was calculated by adjusting the scale for each image [21]. Finally, artifacts were removed, image contrast increased, and the edges of regions of interest were revealed by Gaussian and Laplacian filters [22]. Next, the images were passed over a Gaussian noise filter by removing high-frequency data and passing (retaining) the low-frequency data. It is worth mentioning that the Gaussian filter smoothing factor is a linear low-frequency spatial filter for blurred images and noise removal. Equation (1) shows how a Gaussian filter works.

(1) $$h\left(x\right)=\frac{1}{\sigma \surd 2\pi \;}\;e\;\frac{-{\left(x-\mu \right)}^{2}}{2\sigma^{2}}$$

where μ represents the mean of *x*, and σ represents the standard deviation of *x*.

Thereafter, the images were passed to a Laplacian filter to show the edges of the lesion in the images of pathological tissue, as formulated in Equation (2).

(2) $$\nabla {\;}^{2}\;f=\frac{d\;f}{d^{\;2}\;x}+\frac{d{\;}^{2}\;f}{d^{\;2}\;y}$$

where *x* and *y* represent the coordinates of the pixels in the image.

In the end, the outputs of the filters were overlapped to produce an enhanced image by subtracting the Gaussian filter output from the enhanced Laplacian input to enhance histopathological images, as illustrated in Equation (3).

(3) $$O\left(X\right)=h\left(x\right)-\nabla {\;}^{2}\;f$$

A set of optimized pathological images are shown in Figure 2b after the enhancement process including removing noise, increasing the contrast, and revealing the edges of the area of interest.

### 3.3. Hybrid of CNN and SVM

In this section, we present a novel methodology hybridizing the CNN and SVM algorithms. The rationale behind this hybridization is to overcome the challenges of both computational resources drain and the slowness of CNN models. As a result of hybridization, these challenges can be solved as it requires a low-cost computer, enables fast training of the data set, and yields highly efficient diagnostic results. This hybrid method consists of two parts: The first part is CNN models (AlexNet and ResNet-18) that receive histological images of OSCC after the enhancement process and extract deep feature maps, store them in feature vectors, and send them to the second part, which is the SVM algorithm. SVM replaces the last layers in CNN models. SVM receives deep feature maps and classifies them by classifying each feature vector into its correct class [23].

#### 3.3.1. Extracting Deep Features Maps

The superior ability of CNN models to extract deep feature maps sets them apart from other artificial intelligence technologies. During the training stage, CNN models extract features to classify those extracted during the test stage. Many layers and levels extract deep features, and therefore, each layer is responsible for extracting specific features; for example, the first layer extracts color features, the second layer extracts engineering features, the third layer extracts the features of the texture, and so on; each layer has a specific task [24]. Additionally, in CNN models, a variety of layers exist, and each layer differs from the others. The most important CNN layers are the convolutional layers followed by auxiliary layers and pooling layers followed by auxiliary layers and fully connected layers. The paragraphs below show a brief explanation of the three layers.

**Convolutional layers**: Convolutional layers are one of the critical layers of CNN models, the number of which varies from one model to another. Three main parameters control how convolutional layers work: filter size, zero padding, and P-Step [25]. Filter size determines the number of pixels *f(t)* which wraps around the same number of pixels in target image *x(t)*. Zero padding preserves the size of the original image. The filter moves on the image based on the P-Step. For example, if P-Step = 1, the filter moves by 1, while if P-Step = 2, the filter moves by 2. Equation (4) describes the process of wrapping the filter around the image.

(4) z(t)=(x∗f)(t)=∫x(a)f(t−a)  da

where *f (t)* is a filter, *x(t)* is the input, and *z(t)* refers to the output.

**Pooling layer**: Because of the millions of parameters, connections, and neurons produced by convolutional layers, this is a challenge for CNN models due to the complex computational processes. Thus, CNN models solve this challenge using pooling layers that reduce the dimensions of the images resulting from the convolutional layers. Image dimensions are reduced according to two methods: max and average pooling. Each method has a specific mechanism for reducing dimensions. First, the max-pooling method selects a set of pixels in the target image based on the filter size, selects the max pixel from the selected pixels, and replaces all the pixels chosen with one max pixel as in Equation (5). Secondly, the average pooling method selects a set of pixels in the target image based on the filter size, and it works on calculating the average of all the selected pixels. Then, it replaces all the selected pixels with one pixel representing the selected pixels’ average as in Equation (6).

(5) $$z\left(i;\;j\right)=max_{m,n=1\ldots k}\;f\left[\left(i-1\right)p+m;\;\left(\;j-1\right)p+n\right]\;\;\;\;$$

(6) $$z\left(i;\;j\right)=\frac{1}{k^{2}}\underset{m,n=1\ldots k}{\sum }f\left[\left(i-1\right)p+m;\;\left(\;j-1\right)p+n\right]\;$$

where *f* is the pixels in the filter; *m*, *n* are the dimensions of the image; *k* is the image size; and *p* is the step.

**Auxiliary layers**: the CNN models also contain auxiliary layers such as the rectified linear unit (RLU) that follow some convolutional layers. The RLU layer passes the positive values while denying negative values and converting them to zero. Equation (7) shows how the RLU layer works.

(7) $$\mathrm{ReLU}\left(x\right)=\max\left(\;0,\;x\;\right)=\{\begin{array}{c} x,\;\;\;\;\;\;\;\;\;x\geq 0\\ 0,\;\;\;\;\;\;\;\;\;x<0 \end{array}$$

In this section, deep feature maps were extracted by the AlexNet [26] and Resnet-18 [27] models and stored in the vector features to be sent to the machine learning algorithm to classify them.

CNN models extract high-dimensional features, and therefore, the PCA algorithm was applied to reduce the dimensionality of the data set.

#### 3.3.2. Support Vector Machine

The SVM algorithm replaces the last layers in CNN models. The SVM receives the deep features extracted by AlexNet and ResNet-18 and diagnoses them with high accuracy and less training time. 

SVM first sets all the values of the data set in the n-dimensional space, as n represents the data set’s features [28]. Then, every value of the data set features is represented in absolute coordinates. Consequently, the algorithm works to create many breaks (lines) between the values of the classes called hyperplanes, and the algorithm chooses the best hyperplane with the maximum margin among the classes. Hence, the algorithm can classify any new data point efficiently where it selects the best points that help it choose the appropriate hyperplane. These points are located near or on the hyperplane, called a support vector. The SVM algorithms have two types, linear and non-linear. When the data set is linearly separable, then linear SVM is applied. While if the data set is non-linearly separable, the non-linear SVM is used. In this work, the data set was separated into two classes by the linear SVM algorithm [29].

**Figure 3:**
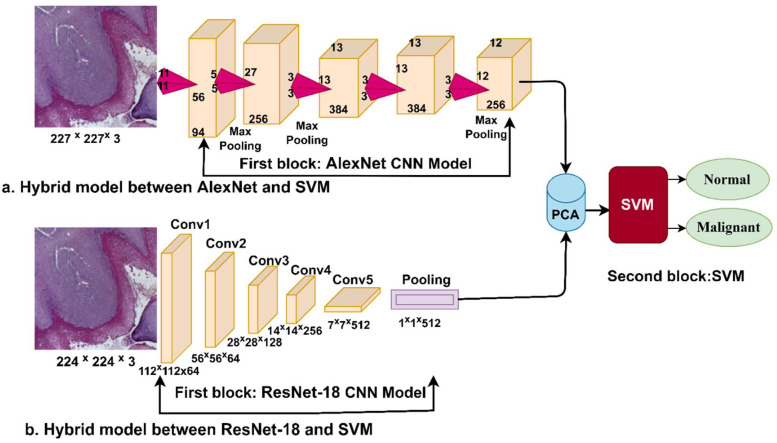
Methodology for histopathological image diagnosis of oral cancer by a hybrid technique between CNN and SVM models.

Figure 3 shows the hybrid technique for diagnosing the pathological images of OSCC. The CNN models are applied to extract deep features, store them in the vectors of features, and send them to the PCA algorithm to reduce dimensions [30]. Finally, low-dimensions features are sent to the SVM algorithm for diagnosing them with high accuracy.

### 3.4. ANN Based on the Hybrid of Deep Features and Traditional Algorithms

This section diagnoses histopathological images of oral cancer by extracting hybrid features using AlexNet and ResNet-18 models, fusing them with features of traditional algorithms (DWT, LBP, FCH, and GLCM), then feeding the hybrid features to the ANN network for classification with high accuracy [31]. It is worth noting that this method is fast in training the data set.

The proposed method works as follows: First, all histopathological images of OSCC are enhanced and then fed to CNN models. All histopathological images are processed through CNN layers to extract deep feature maps for each image and store them in feature vectors. CNN models produced 4096 representative features for each image. The features are stored in feature vectors. Thus, the size of the data set becomes 5192 × 4096.

Second, it is noted that each histological image is represented by 4096 features, and therefore, the size of the data set is high dimensional. Thus, the PCA algorithm was used, which reduces the dimensions of the data set and preserves the essential features in feature vectors. Therefore, after applying the PCA algorithm, the size of the data set becomes 5192 × 1024.

Third, after the histopathological images were subjected to enhancement, the most crucial representative features were extracted by four hybrid algorithms: DWT, LBP, FCH, and GLCM. Shape, color, and texture are the essential features for obtaining high classification accuracy. The DWT algorithm extracts 12 features by analyzing the input signals based on low- and high-pass filters. Low filters produce approximation parameters, while high filters produce three detailed parameters (horizontal, vertical, and diagonal). Thus, each filter extracts three features: the mean, the variance, and the standard deviation. Therefore, the total features extracted by the DWT algorithm are 12 features.

The LBP algorithm extracts the texture features of the binary surfaces by measuring the contrast of local pixels and the pattern of local texture pixels. The algorithm works to change each pixel of the image according to the neighboring pixels, where the algorithm is set to 5 * 5 pixels. Therefore, each target pixel is replaced by 24 adjacent pixels according to Equation (8). The algorithm compares the density of the gray levels of the target pixel (*g_c_*) and the pixels adjacent to it (*g_p_*) [32].

(8) $$LBP_{R,P}=\overset{P-1}{\underset{p=0}{\sum }}s\left(g_{p}-g_{c}\right)2^{p}\;\;\;$$

where *R* denotes the radius for adjacent, $g_{p}$ denotes the gray weight of adjacent pixels, $g_{c}$ denotes the gray weight of the object pixel (central), and *P* is the number of adjacent pixels.

Thus, the LBP algorithm has the ability to distinguish pixels by examining the image density and comparing each pixel with the neighboring. The LBP 203 produces a representative texture feature.

FCH algorithm for color features extraction. Color is one of the essential features for classifying histopathological images. Each local color is represented in the histogram bin, and thus the colors of the target area are distributed in the histogram bin. The two colors in the same bin are similar, while when they are in different bins, the two colors are different even if the two colors are similar. The FCH algorithm compares the similarity of colors through the membership value of each pixel and its distribution over the total histogram bin [33]. The FCH algorithm extracts sixteen color features for each histopathological image of OSCC.

The GLCM algorithm is an array containing different gray levels of the region of interest. GLCM extracts texture features based on the co-occurrence matrix of gray levels. The region of interest contains smooth and coarse regions. When the pixels of the region are close together, the region is smooth, while when the pixels of the region are significantly different, the region is rough. GLCM collects spatial information to calculate statistical texture metrics. Spatial information determines the relationship between pairs of pixels based on distance d and direction θ and describes the location of each pixel from the other. Each pixel is determined from the other by the four values of the directions θ: 0°, 45°, 90°, and 135°; the directions are controlled by the distance where when θ = 0 or θ = 90, the distance d = 1, while when it is = 45 or θ = 135, the distance between one pixel and the other is d = √2. The GLCM algorithm produces 13 statistical features [34].

Fourth, all features extracted from CNN models (AlexNet and ResNet-18) are fused with features extracted by the DWT, LBP, FCH, and GLCM algorithms. After the merge operation, the size of the data set becomes 5192 × 1268.

The feature matrix is fed to the ANN for classification. The ANN consists of input layers by 1268 input units and ten hidden layers for performing complex calculations for solving classification problems. The excretory layer consists of two neurons to sort each image as either normal or malignant.

**Figure 4:**
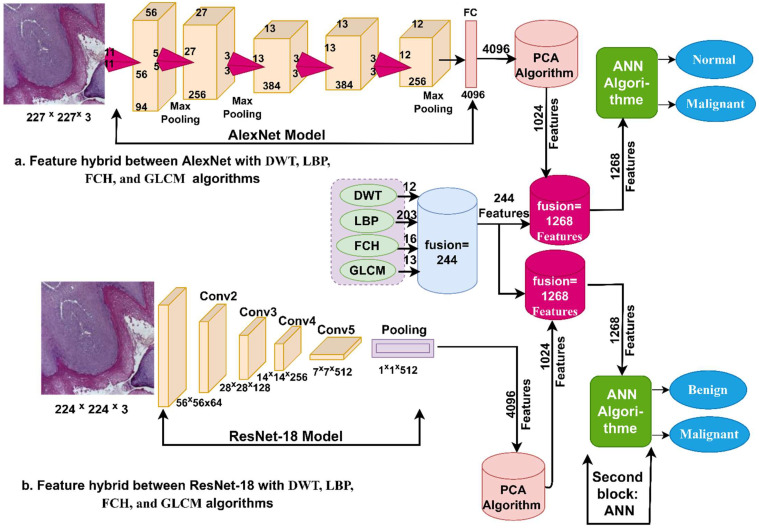
Methodology for histopathological image diagnosis of oral cancer using the hybrid features technique between CNN model, DWT, LBP, FCH, and GLCM models.

Figure 4 illustrates the basic methodology of the proposed method for extracting histopathological features using AlexNet and ResNet-18 models and combining them with features extracted by the DWT, LBP, FCH, and GLCM algorithms. This method is considered a novelty and one of the main contributions of this study, which achieved impressive results for diagnosing histopathological images of OSCC.

### 3.5. The ANN Based on CNN Features

**Figure 5:**
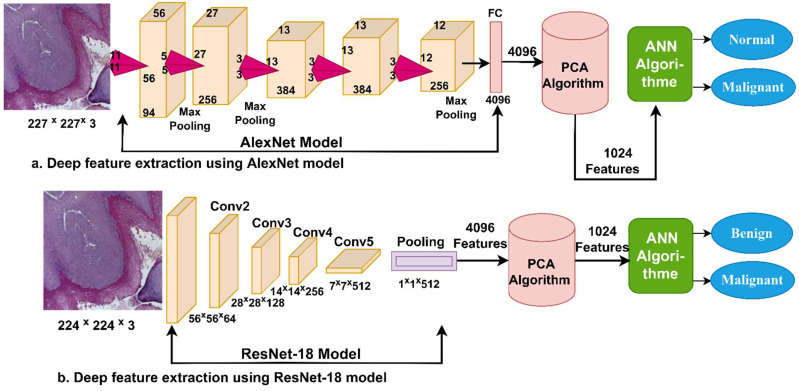
Methodology for histopathological image diagnosis of oral cancer using ANN based on deep feature AlexNet and ResNet-18 models.

This section discusses the diagnosis of the histopathological images of an oral cancer data set by the ANN algorithm based on deep feature extraction using AlexNet and ResNet-18 models. The steps of this method are as follows: First, the histopathological images were optimized to remove noise and increase the contrast of the region of interest and then fed to AlexNet and ResNet-18 models. Second, AlexNet and ResNet-18 models analyzed the input images, extracted deep features by convolutional layers, and stored them in feature vectors with the size of 5192 × 4096 for AlexNet and ResNet-18 models separately. Third, because of the high-dimensional features, the PCA algorithm was applied after feature extraction by AlexNet and ResNet-18 models to reduce the high-dimensional features. Thus, the high-dimensional feature vectors were reduced to become the size of 5192 × 1024 for both AlexNet and ResNet-18 models separately. Finally, low-dimensional feature vectors were fed to the ANN algorithm to classify them into two classes, OSCC and normal (non-OSCC), as shown in Figure 5.

## 4. The Results of the Proposed Systems

### 4.1. Split the Data Set

This study aimed to classify histological images for early diagnosis of oral OSCC by modern methodologies based on hybrid techniques between CNN models and machine learning algorithms, feature extraction, and fusion. The OSCC data set contains 5192 histological images obtained by biopsy and is divided into two classes as follows: 2494 normal histological images and 2698 malignant histological images. The data set was randomly divided into 20:80: 80% during the training and validation phase and 20% for the testing phase. Table 1 shows the split of the data set over all phases of the system. It is worth noting that this division is equal throughout all the proposed methods in this study.

### 4.2. Evaluation of the Proposed Systems

Two proposed methods were used in this study, each with two different systems. All the proposed systems in this study were evaluated with the same criteria. All the systems produced a confusion matrix through which the system evaluation criteria were calculated: accuracy, specificity, sensitivity, precision, and AUC, shown in Equations (9)–(13) [35]. Looking at the equations shown, TP and TN are the numbers of histological images that are correctly classified, representing the primary diameter in the confusion matrix. FP and FN are the numbers of histological images incorrectly classified representing the remainder of the confusion matrix cells.

(9) $$\mathrm{Accuracy}=\frac{\mathrm{TN}+\mathrm{TP}}{\mathrm{TN}+\mathrm{TP}+\mathrm{FN}+\mathrm{FP}}\times 100\%$$

(10) $$\mathrm{Specificity}=\frac{\mathrm{TN}}{\mathrm{TN}+\mathrm{FP}}\times 100$$

(11) $$\mathrm{Sensitivity}=\frac{\mathrm{TP}}{\mathrm{TP}+\mathrm{FN}}\times 100\%$$

(12) $$\mathrm{Precision}=\frac{\mathrm{TP}}{\mathrm{TP}+\mathrm{FP}}\times 100\%$$

(13) $$\mathrm{AUC}\;=\frac{\mathrm{True}\;\mathrm{Positive}\;\mathrm{Rate}}{\mathrm{False}\;\mathrm{Positive}\;\mathrm{Rate}}=\frac{\mathrm{Sensitivity}}{\mathrm{Specificity}}$$

where:

TP is images correctly classified as malignant. TN is images correctly classified as normal. FP are normal images classified as malignant. FN is the malignant images classified as normal.

### 4.3. Data Augmentation Technique

All proposed systems were evaluated on the OSCC data set, consisting of two classes: normal histopathology representing 48% of the data set and histopathological images of malignant tumors representing 52% of the data set. Moreover, CNN models require a huge data set during the training phase to obtain promising results and prevent overfitting problems. Therefore, the data set does not contain a sufficient number of images to train the data set and is somewhat balanced; despite the number of images in the data set classes being close, the data augmentation technique was applied for two purposes: First, to increase the histological images of the data set during the training phase to overcome overfitting problems [36]. Second, to address the issue of imbalance of the data set by increasing the histological images of the minority classes more than the classes of the majority. There are many methods used by the data augmentation method, such as multi-angle rotation, flipping, shifting, etc. Table 2 shows the number of samples for the data set during the training phase before and after data augmentation.

### 4.4. Experimental Results of the Hybrid Method between CNN and SVM

This section presents the experimental results of the proposed hybrid method between CNN models (AlexNet and ResNet-18) and the SVM algorithm. Because CNN models take a long time during the training phase, the classification layers were removed from CNN models and replaced with SVM. Hence, the proposed method consists of two parts: First, CNN models that extract feature maps and store them in feature vectors. The second part is the SVM that receives feature maps and classifies them with high accuracy and at high speed. In this method, two CNN models are applied with the SVM called AlexNet + SVM and ResNet-18 + SVM. Table 3 shows the evaluative performance of hybrid approaches for diagnosing the OSCC data set.

It is noted the ResNet-18 + SVM is superior to AlexNet + SVM, where the AlexNet + SVM achieved an accuracy of 97.4%, specificity of 97.55%, sensitivity of 97.81%, precision of 97.63%, and AUC of 98.25%, while ResNet-18 + SVM achieved accuracy of 98.1%, specificity of 98.35%, sensitivity of 98.61%, precision of 98.22%, and AUC of 97.76%.

**Figure 6:**
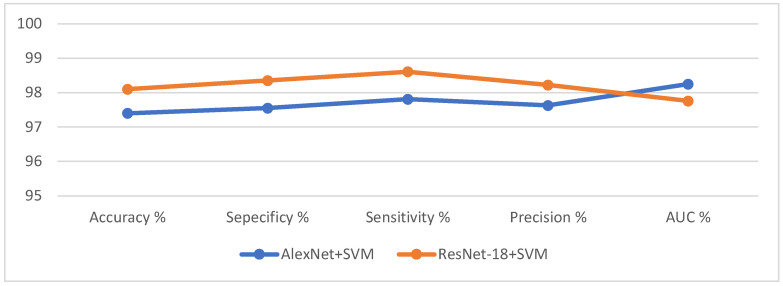
Evaluation of histopathological images for the diagnosis of OSCC.

Figure 6 shows the results of hybrid techniques for histopathological image evaluation for early diagnosis of OSCC. 

**Figure 7:**
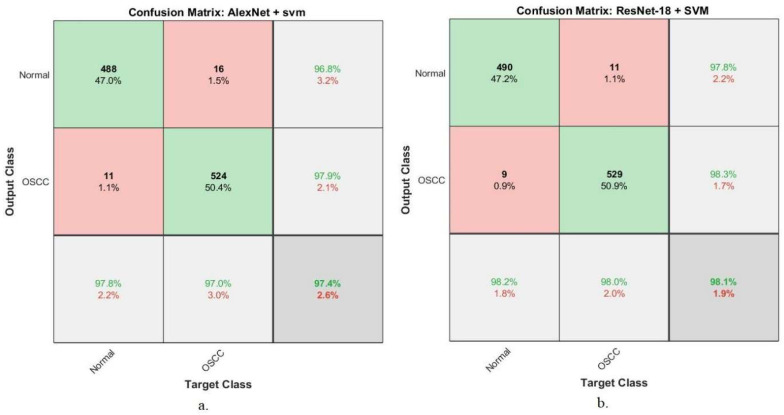
Confusion matrix for performing hybrid techniques for OSCC data set diagnostics. (**a**) AlexNet + SVM; (**b**) ResNet-18 + SVM.

Figure 7 shows the performance of hybrid technologies (AlexNet + SVM and ResNet-18 + SVM) by producing a confusion matrix. AlexNet + SVM achieved an accuracy of 97.8% and 97% for diagnosing normal and OSCC classes, respectively. In contrast, ResNet-18 + SVM achieved an accuracy of 98.2% and 98% for diagnosing normal and OSCC classes, respectively.

### 4.5. The Experimental Results of ANN Based on the Merge Features

This section summarizes the performance of the ANN algorithm for histopathological image diagnosis of OSCC based on the hybrid features extracted from CNN models (AlexNet and ResNet-18) and conventional algorithms (DWT, LBP, FCH, and GLCM). This technique extracted 4096 features from each AlexNet and ResNet-18, then fed them into the PCA algorithm for reduction dimensionality that produced 1024 features for each image, combined with 244 features extracted by traditional algorithms. Thus, after merging all the features, 1268 features were created for each image and fed to the ANN algorithm for classification. The ANN contains input layers consisting of 1268 input units and 15 hidden layers in which all required tasks are solved and an output layer consisting of two neurons, each neuron representing a class of the data set. The section reviews a set of network performance evaluation tools.

#### 4.5.1. Error Histogram

**Figure 8:**
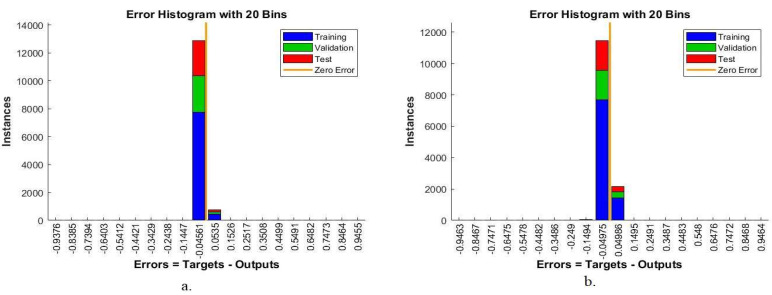
Error histogram for evaluating ANN performance based on hybrid features. (**a**) AlexNet, DWT, LBP, FCH, and GLCM; (**b**) ResNet-18, DWT, LBP, FCH, and GLCM.

The error histogram is one of the ANN performance criteria tools for diagnosing oral squamous cells. This tool measures the error rate between the target values and the output. The network performance for all phases is evaluated by a histogram bin. The network produces a histogram bin in different colors; each color represents a phase, where the blue color represents the network performance during the training phase, the green color represents the network performance during the validation phase, the red color represents the network performance during the testing phase, and finally, the orange color represents the best performance. Figure 8 shows the error histogram produced by the ANN algorithm to evaluate its performance on the OSCC data set. The ANN algorithm based on the hybrid features of AlexNet, DWT, LBP, FCH, and GLCM achieved the best performance with 20 bins ranging from −0.9376 to 0.9455, while the same algorithm based on the hybrid features of ResNet-18, DWT, LBP, FCH, and GLCM achieved the best performance with 20 bins ranging from −0.9463 to 0.9464.

#### 4.5.2. Gradient and Validation Checks

**Figure 9:**
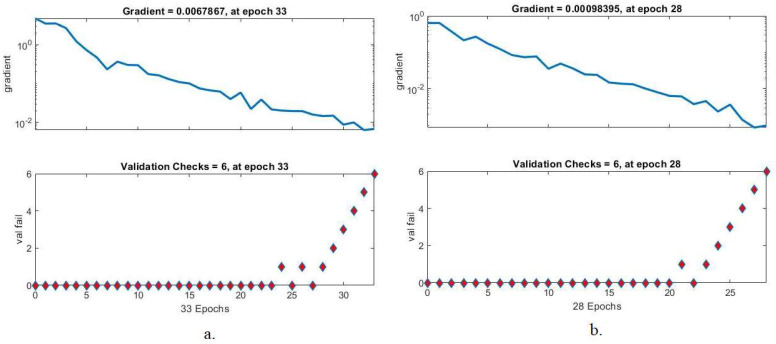
Gradient for evaluating ANN performance based on hybrid features. (**a**) AlexNet, DWT, LBP, FCH, and GLCM; (**b**) ResNet-18, DWT, LBP, FCH, and GLCM.

Gradient and validation checks are one of the ANN’s performance criteria for classifying histological images of OSCC. This tool obtains the best network performance through gradient and validation checks in each epoch that records gradient and validation checks so that the best performance is obtained at the minimum error. Figure 9 shows the gradient and validation checks for the performance of the ANN algorithm for evaluating the OSCC data set. The hybrid feature-based ANN algorithm for AlexNet, DWT, LBP, FCH, and GLCM achieved the best performance at a 0.0067867 gradient and six validations in epoch 33. In contrast, the same hybrid feature-based algorithm for ResNet-18, DWT, LBP, FCH, and GLCM achieved the best performance at the gradient of 0.00098395 and six validations at epoch 28.

#### 4.5.3. Receiver Operating Characteristic (ROC)

**Figure 10:**
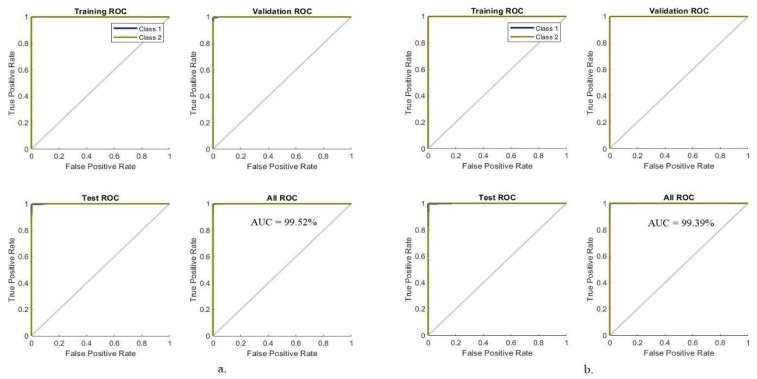
ROC for evaluating ANN performance based on hybrid features. (**a**) AlexNet, DWT, LBP, FCH, and GLCM; (**b**) ResNet-18, DWT, LBP, FCH, and GLCM.

ROC is one of the most important criteria for evaluating the performance of the ANN for classifying histological images of OSCC. The ROC measures false positives represented by the *x*-axis and true positive samples represented by the *y*-axis, which is called AUC. The network performance was evaluated during all phases; in each phase, the AUC is calculated by dividing the true positive rate by the false positive rate. Figure 10 shows the AUC produced by the ANN algorithm to evaluate its performance on the OSCC data set. The ANN algorithm based on the hybrid features of AlexNet, DWT, LBP, FCH, and GLCM achieved the best performance with 99.52%, while the same hybrid feature-based algorithm of ResNet-18, DWT, LBP, FCH, and GLCM achieved the best performance with 99.39%.

#### 4.5.4. Best Validation Performance

**Figure 11:**
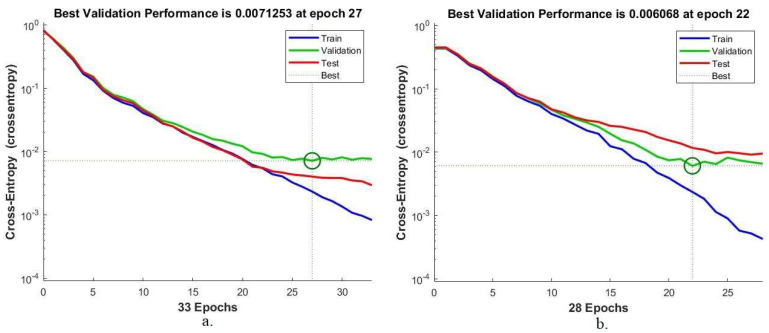
Best validation for evaluating ANN performance based on hybrid features. (**a**) AlexNet, DWT, LBP, FCH, and GLCM; (**b**) ResNet-18, DWT, LBP, FCH, and GLCM.

The mean squared error, or cross-entropy, is one of the most important criteria for evaluating the performance of the ANN network for classifying histological images of OSCC. This tool measures the error rate between the actual expected values. The ANN evaluates the data set during all phases. The network produces cross-entropy in different colors; each color represents a specific stage, where the blue color represents the network performance during the training phase, the green color represents the network performance during the verification phase, the red color represents the network performance during the testing phase, and finally, the dashed lines represent the best network performance. Figure 11 shows the cross-entropy of the ANN algorithm to evaluate its performance on the OSCC data set. The ANN algorithm based on the hybrid features of AlexNet, DWT, LBP, FCH, and GLCM achieved the best performance when reaching a minimum error of 0.0071253 at epoch 27. The same algorithm based on the hybrid features of ResNet-18, DWT, LBP, FCH, and GLCM achieved the best performance when reaching the minimum error of 0.006068 at epoch 22.

#### 4.5.5. Confusion Matrix

**Figure 12:**
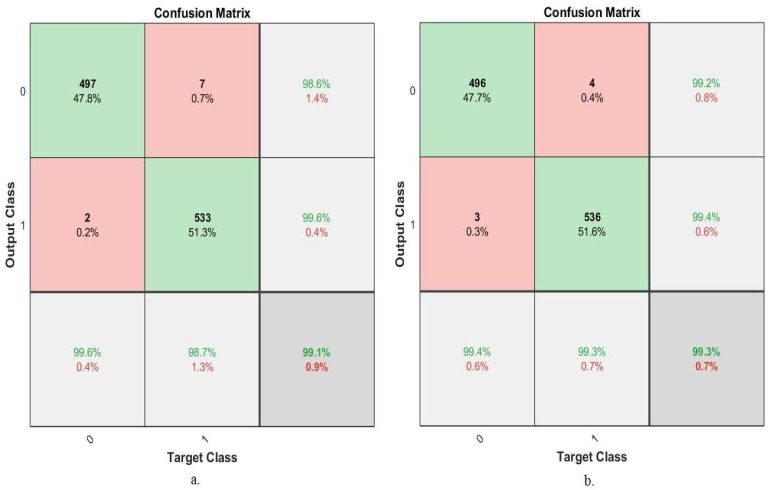
Confusion matrix for evaluating ANN performance based on hybrid features. (**a**) AlexNet, DWT, LBP, FCH, and GLCM; (**b**) ResNet-18, DWT, LBP, FCH, and GLCM.

The confusion matrix is the essential criterion for evaluating the performance of all proposed systems for histological image diagnosis of OSCC. A confusion matrix is a form of a quaternary matrix (the number of rows equals the number of columns), containing all images of the data set that are correctly classified, called TP and TN, and all images that are incorrectly classified, called FP and FN. Correctly sorted images fall on the matrix’s main diagonal, while incorrectly sorted images fall into the rest of the confusion matrix cells. Figure 12 shows the confusion matrix produced using the ANN algorithm to evaluate performance on the OSCC data set. Class 1 represents a normal class, and class 2 represents a malignant class (OSCC). The ANN algorithm based on the hybrid features of AlexNet, DWT, LBP, FCH, and GLCM achieved an overall accuracy of 99.1%, while the same hybrid feature-based algorithm of ResNet-18, DWT, LBP, FCH, and GLCM achieved an overall accuracy of 99.3%.

The hybrid features extracted from the CNN models and traditional algorithms contributed to promising results in the histological image diagnosis of OSCC. Table 4 describes the performance of the ANN based on the hybrid features, which yielded promising results. The ANN algorithm based on the hybrid features of AlexNet, DWT, LBP, FCH, and GLCM achieved an accuracy of 99.1%, specificity of 99.61%, sensitivity of 99.5%, precision of 99.71%, and AUC of 99.52%, while the same algorithm based on the hybrid features of ResNet-18, DWT, LBP, FCH, and GLCM achieved an accuracy of 99.3%, specificity of 99.42%, sensitivity of 99.26%, precision of 99.31%, and AUC of 99.39%.

**Figure 13:**
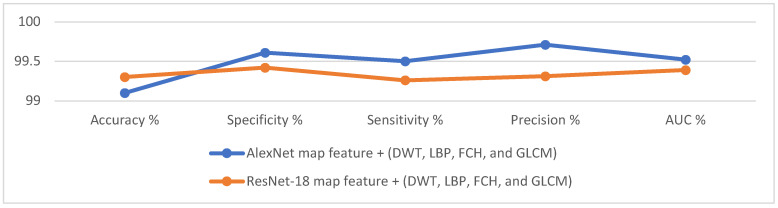
ANN performance based on the hybrid features between CNN models and traditional algorithms.

Figure 13 presents the evaluative performance of the ANN for histological image diagnosis for early diagnosis of OSCC.

### 4.6. The Result of ANN Based on CNN Features

This section discusses the results of ANN performance based on deep features extracted from AlexNet and ResNet-18 models for histopathological diagnosis of an oral cancer data set. The proposed method consists of two parts: the first part is an AlexNet and ResNet-18 model for deep feature extraction, and the second part is an ANN network for deep feature diagnosis. The performance results of the AlexNet + ANN and ResNet-18 + ANN techniques for OSCC data set diagnostics are shown in Table 5. It is noted that the ANN with AlexNet model features is superior to the ResNet-18 model features. An ANN with AlexNet model features achieved an accuracy of 96.5%, specificity of 96.46%, sensitivity of 96.31%, precision of 96.87%, and AUC of 97.57%. In contrast, an ANN with ResNet-18 model features achieved an accuracy of 95.1%, specificity of 95.28%, sensitivity of 94.84%, precision of 94.97%, and AUC of 97.12%.

**Figure 14:**
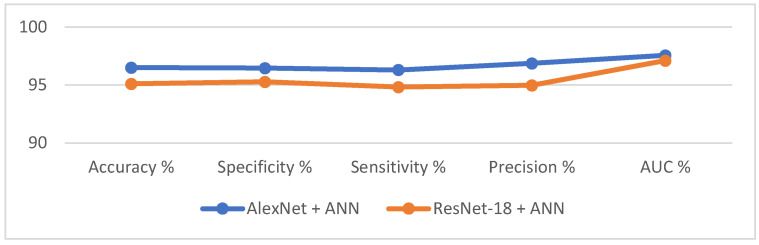
Display of ANN performance based on the features of AlexNet and ResNet-18 models.

Figure 14 shows the results of ANN performance based on deep features extracted by AlexNet and ResNet-18 models.

**Figure 15:**
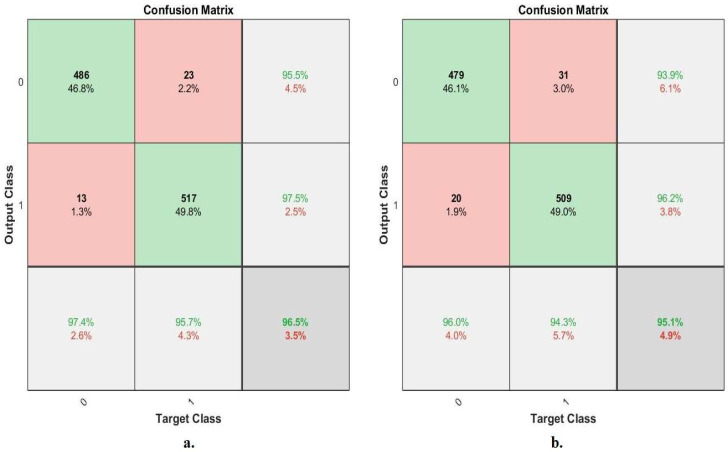
Confusion matrix for OSCC data set diagnosis by ANN based on deep features of models. (**a**) AlexNet; (**b**) ResNet-18.

Figure 15 shows the performance of ANN based on the features extracted by AlexNet and ResNet-18 models after high dimensionality reduction by the PCA algorithm for OSCC data set diagnosis. Based on AlexNet features, the ANN achieved 100% overall accuracy, diagnostic accuracy for the OSCC class 100%, and diagnostic accuracy for the normal class 100%. In contrast, the ANN based on ResNet-18 features achieved an overall accuracy of 100%, an accuracy for the OSCC class of 100%, and for the normal class, an accuracy of 100%.

## 5. Discussion of the Proposed Methods

This study discussed modern methods for early diagnosis of OSCC through two proposed methods, each of which has two systems with different methodologies. All OSCC data set images were optimized by two filters; it is worth noting that the same two filters were used for all the proposed systems. Due to the lack of OSCC images, which causes overfitting, the data augmentation method was applied to increase the data set images artificially. The proposed methods are discussed as follows: The first proposed method is a hybrid method consisting of two approaches, namely, CNN models (AlexNet and ResNet-18), whose task is to extract the features and then reduce the dimensions by the PCA algorithm and store them in feature vectors, and the SVM algorithm, whose task is to receive and classify CNN feature vectors with high speed and accuracy. The second proposed method is to classify the OSCC data set based on hybrid features extracted by the CNN, DWT, LBP, FCH, and GLCM.

The first proposed system represents one of our contributions in this work. Classification layers were removed from the AlexNet and ResNet-18 models and replaced with the SVM algorithm. This technique solves some of the problems of CNN models, such as the time consumed when training the data set and the need for a high-performance and expensive computer. Thus, this method is quick to implement and train the data set on a medium-cost computer. AlexNet+SVM and ResNet-18 achieved an overall accuracy of 97.4% and 98.1%, respectively. The second proposed method, one of our contributions and novelty, is an ANN network based on the hybrid features extracted by CNN models and combined with the features of DWT, LBP, FCH, and GLCM algorithms. The CNN features were extracted and dimensionally reduced by the PCA algorithm and then combined with the features of DWT, LBP, FCH, and GLCM. An ANN based on AlexNet, DWT, LBP, FCH, and GLCM features achieved an overall accuracy of 99.1%, while the same network based on the features of ResNet-18, DWT, LBP, FCH, and GLCM achieved an overall accuracy of 99.3%.

Table 6 describes all the proposed systems for histopathological image diagnosis for early diagnosis of OSCC. It is noted that the table contains the overall accuracy of each system in addition to the accuracy achieved by each system for each class. Here is presented a review of the best diagnostic accuracy for each class. It is noted that the ANN network based on the features extracted by AlexNet, FCH, DWT, LBP, and GLCM reached an accuracy of 99.6% for diagnosing normal histological images. In contrast, the ANN based on the features extracted by ResNet-18, FCH, DWT, LBP, and GLCM achieved an accuracy of 99.3% for diagnosing histological images of malignant tumors.

**Figure 16:**
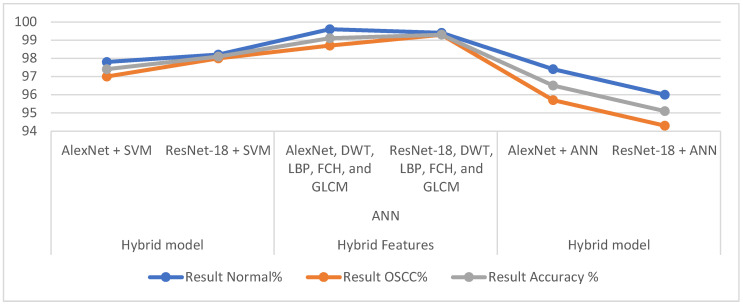
Performance of the proposed methods for OSCC data set diagnostics.

Figure 16 shows the performance of the methods proposed in this study to diagnose the OSCC data set. 

**Figure 17:**
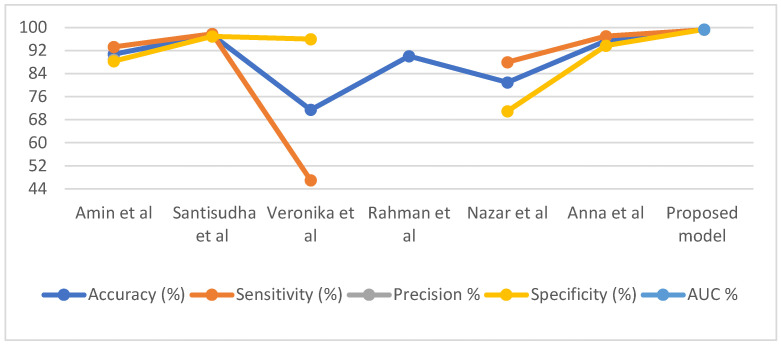
Comparison of the performance of our system with previous studies for diagnosing oral squamous cell carcinomas [6,10,16,37,38,39].

Table 7 and Figure 17 illustrate the performance comparison of the proposed systems achieved with previous studies related to the diagnosis of histopathological images of the oral squamous cell carcinoma data set. It is noted that the performance of our system is superior to the previous studies, and it is noted that our system was evaluated by many evaluation scales compared to the evaluation measures of previous studies that were limited to some measures. Previous studies reached an accuracy of between 81% and 97.35%, while our system achieved an accuracy of 99.3%. Previous studies reached a sensitivity of between 88% and 97.78% while our system achieved a sensitivity of 99.26%. Previous studies reached a specificity of between 71% and 96.92%, while our system achieved a specificity of 99.42%.

## 6. Conclusions and Future Work

Histopathological image analysis is one of the essential methods for diagnosing OSCC based on abnormal tissue. Manual diagnosis depends on the competence and experience of the doctors, as it takes a long time to trace all the tissues in the biopsy taken from the patient. Despite this, the manual diagnosis still has shortcomings and doctors’ differing opinions about the diagnosis. This study highlighted the tremendous potential of artificial intelligence techniques to diagnose OSCC and increase cure rates among patients. This work applied two proposed methods; each method has two systems with different methodologies. Two-part hybrid methods were applied: the first part is CNN models (AlexNet and ResNet-18) to extract the deep features and send them to the PCA algorithm to reduce the dimensionality of the data set features. These features are fed into the second part which is the SVM algorithm to classify them with high accuracy. This technique yielded promising results in diagnosing the OSCC data set. Second, the OSCC data set was diagnosed by an ANN based on the hybrid features extracted from the CNN models and combined with the color, texture, and shape features extracted by the DWT, LBP, FCH, and GLCM algorithms. This method yielded promising results in histological image diagnostics for early diagnosis of OSCC. The ANN algorithm based on the hybrid features by ResNet-18, DWT, LBP, FCH, and GLCM reached an accuracy of 99.3%, specificity of 99.42%, sensitivity of 99.26%, precision of 99.31%, and AUC of 99.39%.

The future work of this study is as follows: generalization of the proposed systems to more than one data set, integration of features extracted from more than one CNN model, and their diagnosis by machine learning algorithms and neural networks.

## Figures and Tables

**Table 1:** Splitting of the OSCC data set through all phases for both classes.

Phase	Training and Validation 80%		Testing 20%
Classes	Training (80%)	Validation (20%)	
Normal	1596	399	499
Malignant	1726	432	540

**Table 2:** Data augmentation for histopathological images to balance the data set during the training phase.

Phase	Training Phase	
Classes	Normal	Malignant
Before augmentation	1596	1726
After augmentation	**19,152**	**20,712**

**Table 3:** Evaluative performance of the hybrid method for early diagnosis of OSCC.

Measure	AlexNet + SVM	ResNet-18 + SVM
Accuracy %	97.4	98.1
Specificity %	97.55	98.35
Sensitivity %	97.81	98.61
Precision %	97.63	98.22
AUC %	98.25	97.76

**Table 4:** Performance of ANN based on fusion features for early diagnosis of OSCC.

Hybrid Features	AlexNet Map Feature + (DWT, LBP, FCH, and GLCM)	ResNet-18 Map Feature + (DWT, LBP, FCH, and GLCM)
Accuracy %	99.1	99.3
Specificity %	99.61	99.42
Sensitivity %	99.5	99.26
Precision %	99.71	99.31
AUC %	99.52	99.39

**Table 5:** Evaluative performance of the ANN based on deep features for early diagnosis of OSCC.

Measure	AlexNet + ANN	ResNet-18 + ANN
Accuracy %	96.5	95.1
Specificity %	96.46	95.28
Sensitivity %	96.31	94.84
Precision %	96.87	94.97
AUC %	97.57	97.12

**Table 6:** Results for all proposed systems for OSCC data set diagnostics.

Methods			Features	Size Vector	Time	Result		
						Normal %	OSCC %	Accuracy %
Hybrid model		AlexNet + SVM	Deep features	5192 × 1024	3 min 36 s	97.8	97	97.4
		ResNet-18 + SVM	Deep features	5192 × 1024	4 min 41 s	98.2	98	98.1
Hybrid Features	ANN	AlexNet, DWT, LBP, FCH, and GLCM	Fused features	5192 × 1268	5 min 16 s	99.6	98.7	99.1
		ResNet-18, DWT, LBP, FCH, and GLCM	Fused features	5192 × 1268	6 min 18 s	99.4	99.3	99.3
Hybrid model		AlexNet + ANN	Deep features	5192 × 1024	4 min 26 s	97.4	95.7	96.5
		ResNet-18 + ANN	Deep features	5192 × 1024	5 min 37 s	96	94.3	95.1

**Table 7:** Comparison of our results with previous related studies.

Previous Studies	Accuracy (%)	Sensitivity (%)	Precision %	Specificity (%)	AUC %
Amin et al. [6]	90.83	93.33	88.88	88.33	-
Santisudha et al. [16]	97.35	97.78	-	96.92	-
Veronika et al. [10]	71.5	47	-	96	-
Rahman et al. [37]	90.06	-	-	-	-
Nazar et al. [38]	81	88	-	71	-
Anna et al. [39]	95.35	97	-	93.7	-
**Proposed model**	**99.3**	**99.26**	**99.31**	**99.42**	**99.39**

## Data Availability

In this study, the data set that supports the results of the proposed systems is available at the link: https://www.kaggle.com/datasets/ashenafifasilkebede/dataset (accessed on 15 February 2022).

## References

[1] Du M., Nair R., Jamieson L., Liu Z., Bi P. (2020). Incidence trends of lip, oral cavity, and pharyngeal cancers: Global burden of disease 1990–2017. J. Dent. Res..

[2] Warnakulasuriya S., Greenspan J.S. (2020). Epidemiology of oral and oropharyngeal cancers. Textbook of Oral Cancer.

[3] Perdomo S., Roa G.M., Brennan P., Forman D., Sierra M.S. (2016). Head and neck cancer burden and preventive measures in Central and South America. Cancer Epidemiol..

[4] Chakraborty D., Natarajan C., Mukherjee A. (2019). Advances in oral cancer detection. Adv. Clin. Chem..

[5] Speight P.M., Khurram S.A., Kujan O. (2018). Oral potentially malignant disorders: Risk of progression to malignancy. Oral Surg. Oral Med. Oral Pathol. Oral Radiol..

[6] Amin I., Zamir H., & Khan F.F. (2021). Histopathological Image Analysis for Oral Squamous Cell Carcinoma classification using concatenated deep learning models. medRxiv.

[7] Rahman T.Y., Mahanta L.B., Choudhury H., Das A.K., Sarma J.D. (2020). Study of morphological and textural features for classification of oral squamous cell carcinoma by traditional machine learning techniques. Cancer Rep..

[8] Camalan S., Mahmood H., Binol H., Araújo A.L.D., Santos-Silva A.R., Vargas P.A., Gurcan M.N. (2021). Convolutional neural network-based clinical predictors of oral dysplasia: Class activation map analysis of deep learning results. Cancers.

[9] Rahman T.Y., Mahanta L.B., Das A.K., Sarma J.D. (2020). Automated oral squamous cell carcinoma identification using shape, texture and color features of whole image strips. Tissue Cell.

[10] Shavlokhova V., Sandhu S., Flechtenmacher C., Koveshazi I., Neumeier F., Padrón-Laso V., Freudlsperger C. (2021). Deep Learning on Oral Squamous Cell Carcinoma Ex Vivo Fluorescent Confocal Microscopy Data: A Feasibility Study. J. Clin. Med..

[11] Bhandari B., Alsadoon A., Prasad P.W.C., Abdullah S., Haddad S. (2020). Deep learning neural network for texture feature extraction in oral cancer: Enhanced loss function. Multimed. Tools Appl..

[12] Martino F., Bloisi D.D., Pennisi A., Fawakherji M., Ilardi G., Russo D., Merolla F. (2020). Deep learning-based pixel-wise lesion segmentation on oral squamous cell carcinoma images. Appl. Sci..

[13] Halicek M., Dormer J.D., Little J.V., Chen A.Y., Myers L., Sumer B.D., Fei B. (2019). Hyperspectral imaging of head and neck squamous cell carcinoma for cancer margin detection in surgical specimens from 102 patients using deep learning. Cancers.

[14] Musulin J., Štifanić D., Zulijani A., Ćabov T., Dekanić A., Car Z. (2021). An enhanced histopathology analysis: An ai-based system for multiclass grading of oral squamous cell carcinoma and segmenting of epithelial and stromal tissue. Cancers.

[15] Paderno A., Piazza C., Del Bon F., Lancini D., Tanagli S., Deganello A., Moccia S. (2021). Deep learning for automatic segmentation of oral and oropharyngeal cancer using narrow band imaging: Preliminary experience in a clinical perspective. Front. Oncol..

[16] Panigrahi S., Das J., Swarnkar T. (2020). Capsule network-based analysis of histopathological images of oral squamous cell carcinoma. J. King Saud Univ.-Comput. Inf. Sci..

[17] Bur A.M., Holcomb A., Goodwin S., Woodroof J., Karadaghy O., Shnayder Y., Shew M. (2019). Machine learning to predict occult nodal metastasis in early oral squamous cell carcinoma. Oral Oncol..

[18] Yu M., Yan H., Xia J., Zhu L., Zhang T., Zhu Z., Dong M. (2019). Deep convolutional neural networks for tongue squamous cell carcinoma classification using Raman spectroscopy. Photodiagn. Photodyn. Ther..

[19] Gupta R.K., Kaur M., Manhas J. (2019). Tissue level based deep learning framework for early detection of dysplasia in oral squamous epithelium. J. Multimed. Inf. Syst..

[20] Histopathologic Oral Cancer Detection Using CNNs/Kaggle. https://www.kaggle.com/datasets/ashenafifasilkebede/dataset.

[21] Abunadi I., Senan E.M. (2021). Deep learning and machine learning techniques of diagnosis dermoscopy images for early detection of skin diseases. Electronics.

[22] Bruixola G., Remacha E., Jiménez-Pastor A., Dualde D., Viala A., Montón J.V., Cervantes A. (2021). Radiomics and radiogenomics in head and neck squamous cell carcinoma: Potential contribution to patient management and challenges. Cancer Treat. Rev..

[23] Mohammed B.A., Senan E.M., Rassem T.H., Makbol N.M., Alanazi A.A., Al-Mekhlafi Z.G., Ghaleb F.A. (2021). Multi-method analysis of medical records and MRI images for early diagnosis of dementia and Alzheimer’s disease based on deep learning and hybrid methods. Electronics.

[24] Chu C.S., Lee N.P., Ho J.W., Choi S.W., Thomson P.J. (2021). Deep learning for clinical image analyses in oral squamous cell carcinoma: A review. JAMA Otolaryngol. Head Neck Surg..

[25] El-Hasnony I.M., Elzeki O.M., Alshehri A., Salem H. (2022). Multi-label active learning-based machine learning model for heart disease prediction. Sensors.

[26] Al-Mekhlafi Z.G., Senan E.M., Rassem T.H., Mohammed B.A., Makbol N.M., Alanazi A.A., Ghaleb F.A. (2022). Deep Learning and Machine Learning for Early Detection of Stroke and Haemorrhage. Computers. Mater. Contin..

[27] Ahmed I.A., Senan E.M., Rassem T.H., Ali M.A., Shatnawi H.S.A., Alwazer S.M., Alshahrani M. (2022). Eye Tracking-Based Diagnosis and Early Detection of Autism Spectrum Disorder Using Machine Learning and Deep Learning Techniques. Electronics.

[28] ElAraby M.E., Elzeki O.M., Shams M.Y., Mahmoud A., Salem H. (2022). A novel Gray-Scale spatial exploitation learning Net for COVID-19 by crawling Internet resources. Biomed. Signal Process. Control.

[29] Senan E.M., Jadhav M.E., Rassem T.H., Aljaloud A.S., Mohammed B.A., Al-Mekhlafi Z.G. (2022). Early Diagnosis of Brain Tumour MRI Images Using Hybrid Techniques between Deep and Machine Learning. Comput. Math. Methods Med..

[30] Yang T., Hui R., Nouws J., Sauler M., Zeng T., Wu Q. (2022). Untargeted metabolomics analysis of esophageal squamous cell cancer progression. J. Transl. Med..

[31] Abunadi I., Senan E.M. (2022). Multi-Method Diagnosis of Blood Microscopic Sample for Early Detection of Acute Lymphoblastic Leukemia Based on Deep Learning and Hybrid Techniques. Sensors.

[32] Senan E.M., Jadhav M.E. (2020). Techniques for the Detection of Skin Lesions in PH^2^ Dermoscopy Images Using Local Binary Pattern (LBP). International Conference on Recent Trends in Image Processing and Pattern Recognition.

[33] Senan E.M., Jadhav M.E., Kadam A. Classification of PH2 images for early detection of skin diseases. Proceedings of the 2021 6th International Conference for Convergence in Technology.

[34] Senan E.M., Jadhav M.E. (2022). Diagnosis of dermoscopy images for the detection of skin lesions using SVM and KNN. Proceedings of the Third International Conference on Sustainable Computing.

[35] Senan E.M., Abunadi I., Jadhav M.E., Fati S.M. (2021). Score and Correlation Coefficient-Based Feature Selection for Predicting Heart Failure Diagnosis by Using Machine Learning Algorithms. Comput. Math. Methods Med..

[36] Fati S.M., Senan E.M., ElHakim N. (2022). Deep and Hybrid Learning Technique for Early Detection of Tuberculosis Based on X-ray Images Using Feature Fusion. Appl. Sci..

[37] Rahman A., Alqahtani A., Aldhafferi N., Nasir M.U., Khan M.F., Khan M.A., Mosavi A. (2022). Histopathologic Oral Cancer Prediction Using Oral Squamous Cell Carcinoma Biopsy Empowered with Transfer Learning. Sensors.

[38] Mohamed N., van de Goor R., El-Sheikh M., Elrayah O., Osman T., Nginamau E.S., Johannessen A.C., Suleiman A., Costea D.E., Kross K.W. (2021). Feasibility of a Portable Electronic Nose for Detection of Oral Squamous Cell Carcinoma in Sudan. Healthcare.

[39] Starzyńska A., Sobocki B.K., Sejda A., Sakowicz-Burkiewicz M., Szot O., Jereczek-Fossa B.A. (2021). ZNF-281 as the Potential Diagnostic Marker of Oral Squamous Cell Carcinoma. Cancers.

